# Can Antiviral Activity of Licorice Help Fight COVID-19 Infection?

**DOI:** 10.3390/biom11060855

**Published:** 2021-06-08

**Authors:** Luisa Diomede, Marten Beeg, Alessio Gamba, Oscar Fumagalli, Marco Gobbi, Mario Salmona

**Affiliations:** 1 Department of Molecular Biochemistry and Pharmacology, Istituto di Ricerche Farmacologiche Mario Negri IRCCS, Via Mario Negri 2, 20156 Milano, Italy; marten.beeg@marionegri.it (M.B.); oscar.fumagalli@marionegri.it (O.F.); marco.gobbi@marionegri.it (M.G.); 2 Department of Environmental Health Science, Istituto di Ricerche Farmacologiche Mario Negri IRCCS, Via Mario Negri 2, 20156 Milano, Italy; alessio.gamba@marionegri.it

**Keywords:** licorice, liquorice, glycyrrhizin, glycyrrhizic acid, glycyrrhetinic acid, enoxolone, virus, SARS-CoV-2, COVID-19

## Abstract

The phytotherapeutic properties of *Glycyrrhiza glabra* (licorice) extract are mainly attributed to glycyrrhizin (GR) and glycyrrhetinic acid (GA). Among their possible pharmacological actions, the ability to act against viruses belonging to different families, including SARS coronavirus, is particularly important. With the COVID-19 emergency and the urgent need for compounds to counteract the pandemic, the antiviral properties of GR and GA, as pure substances or as components of licorice extract, attracted attention in the last year and supported the launch of two clinical trials. In silico docking studies reported that GR and GA may directly interact with the key players in viral internalization and replication such as angiotensin-converting enzyme 2 (ACE2), spike protein, the host transmembrane serine protease 2, and 3-chymotrypsin-like cysteine protease. In vitro data indicated that GR can interfere with virus entry by directly interacting with ACE2 and spike, with a nonspecific effect on cell and viral membranes. Additional anti-inflammatory and antioxidant effects of GR cannot be excluded. These multiple activities of GR and licorice extract are critically re-assessed in this review, and their possible role against the spread of the SARS-CoV-2 and the features of COVID-19 disease is discussed.

## 1. Introduction

In ancient times, readily available plant extracts were the first sources of “drugs”. The therapeutic use of *Glycyrrhiza glabra* (licorice) and its extracts dates back to the dawn of time. Scholars of the history of medicine date the first information on its medicinal use to over 4000 years ago [1]. Its use has spread thanks to the ease of agricultural cultivation of its fourteen species in many warm and temperate countries. Therefore, its therapeutic use has been proposed by many cultures and ethnic groups. The study of its phytotherapeutic properties reflects the remarkable variety of products with some therapeutic activity present above all in the roots of the plant [2,3,4]. The pathologies for which these extracts have been used in the past are extremely varied and include disorders of the respiratory, gastrointestinal, cardiovascular, and urinary systems. It was also widely used to treat ocular exenteration and neurodegenerative diseases.

Even the toxic effects of these extracts have been duly recorded in tradition. Of interest is the hypothesis on the death of the philosopher Heraclitus of Ephesus, who had decided to eat only herbs and roots in the last part of his life. Diogenes Laertius describes his death as due to hydropsy because of his vast consumption of licorice roots [5].

Only in the last decades, with the development of adequate analytical methods, has it become possible to understand the nature of the molecules present in the extracts. This has had decisive importance in defining the molecules’ therapeutic potential. The knowledge from the “ancient pharmacopeia” has made it possible to identify plants with potential therapeutic activity and describe their properties in a modern, current context.

Among the compounds identified, glycyrrhizic acid or glycyrrhizin (GR) and its metabolites, mainly glycyrrhetinic acid (GA) or enoxolone, are the most important [1]. Their possible pharmacological action includes the particularly important ability to act against viruses belonging to different families, including the SARS coronavirus [6,7]. With the COVID-19 emergency and the urgent need for compounds to ease the pandemic, the antiviral properties of GR and GA, as pure substances or as components of licorice extract, have attracted fresh attention over the last year.

This review critically reassesses these aspects, widely investigated over the last year, and examines the possible utility of GR and GA against the spread of the SARS-CoV-2 virus. We have reviewed the antiviral properties of GR and GA, as pure substances or as components of licorice, from the year 1947 to the date of the search, which involved the following databases: Embase, Medline, and the Cochrane Library. Title, abstract, and keyword searches were made using Boolean search operators by extract, on the basis of a critical systematic analysis of the literature. To retrieve all the pertinent evidence, the search was done on 28 February 2021 with global geographical coverage and time limits on the ProQuest search engine: (“licorice” OR “liquorice” OR “glycyrrhiza glabra” OR “glycyrrhizic acid” OR glycyrrhizin OR “glycyrrhetinic acid” OR “enoxolone”) AND (antiviral OR virus OR hepatitis). A total of 508 studies were identified after adjusting for duplicates, and then analyzed. After applying a pre-selected inclusion and exclusion criteria based on the authors’ own experience, 84 original articles were deemed pertinent for inclusion in this review.

## 2. From Traditional to Medicinal Use of Licorice Extract 

The passage from empirical and anecdotal “pharmacology and therapy” to the identification of each component of the mixture has enabled us to re-evaluate licorice extracts in modern terms and give each component potential pharmacological dignity. The identification in the extracts of licorice roots of molecules with interesting biological activities contributed to its re-evaluation (Table 1). The diversity of molecules described in these extracts makes it challenging to distinguish information on their biological or pharmacological activity from anecdotal indications and scientifically proven ones [2,3,4].

**Figure 1:**
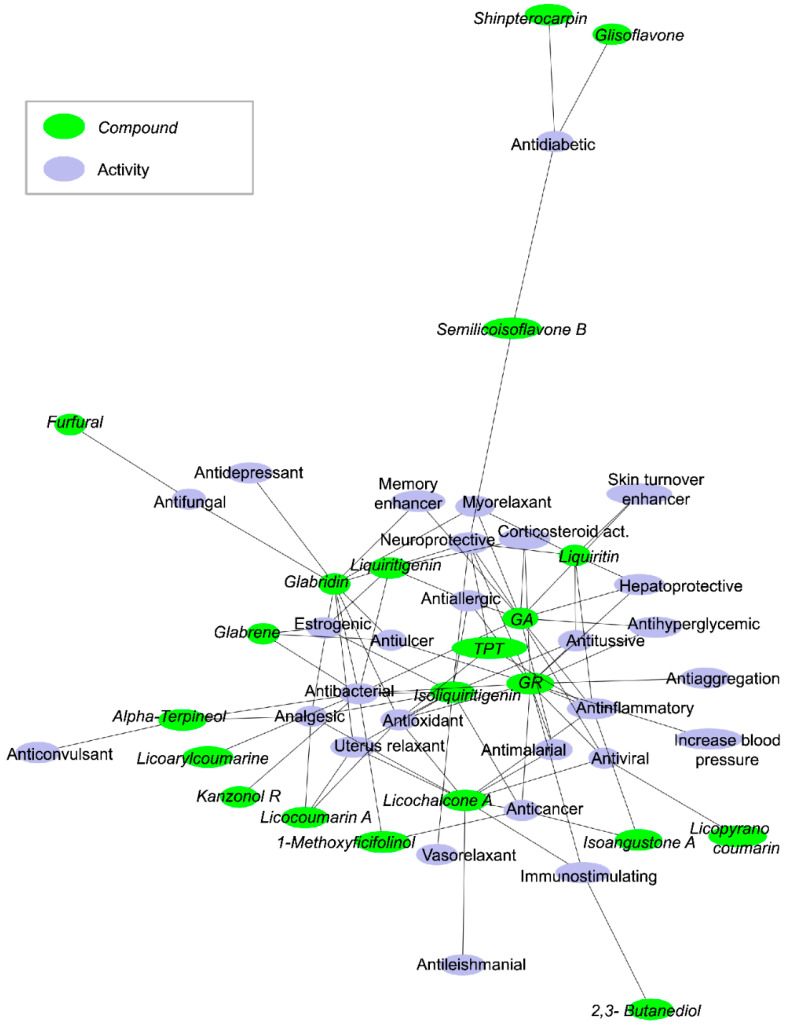
Network of potentially active compounds and their activities in licorice root extracts. The figure illustrates the active compounds (green circles) linked to their pharmacological activities (blue circles). The network is drawn using PyGraphViz and the “fdp” layout. In this representation, the most connected nodes are close together in the center of the network. GA and GR are the compounds with the most activities, and other compounds are on the sides of the network. GA and GR share many activities with other compounds. TPT is tetramethyl pyrazine-2,3,5,6-tetracarboxylate.

We analyzed most of the data in the literature and generated a network of 21 potentially active compounds and 28 biological or pharmacological activities (Figure 1). Many molecules (center of the network) share the same or similar potential activities. Particularly interesting is that the antibacterial activity is one of the primary nodes on which many licorice molecules converge. The most frequent activities are antibacterial (10 connections), neuroprotective (7 connections), antioxidant (6 connections), anti-inflammatory (6 connections), analgesic (5 connections), anticancer (5 connections). The observation that GR or its metabolites have antiviral activity is related to their antioxidant and anti-inflammatory effects, and includes the inhibition of viral internalization, downregulation of pro-inflammatory cytokines, inhibition of the accumulation of intracellular reactive oxygen species (ROS), inhibition of thrombin, inhibition of the hyperproduction of airway exudates, and induction of endogenous interferon (IFN).

Today licorice is widely used as a food ingredient, flavoring agent, and dietary supplement. Licorice and its derivatives are considered “Generally Recognized as Safe” (GRAS) by the U.S. Food and Drug Administration (FDA) [8] and are also approved for use in some over-the-counter drugs [9]. Different limits for licorice consumption have been established by regulatory authorities around the world, based on the GR content. The Joint FAO/WHO Expert Committee on Food Additives (JECFA) indicated that 100 mg/day GR would be unlikely to cause adverse effects in the majority of adults [10]. The Council of Europe and the UK Food Additive and Contaminants Committee established a limit of 50 mg/kg GR [11].

Licorice extracts, GR and GA, have been widely put to medicinal uses and some licorice-containing products are on the market. In Germany, on the basis of traditional use, aqueous licorice extracts alone or in combination with other plant-derived compounds are commercialized to support gastric function and to favor the fluidification of airway mucous [11]. Two aqueous licorice extracts containing 4.64 and 3.92 mg/mL GR have been authorized for more than 70 years as expectorants in Denmark [11].

Importantly, GR is well tolerated. The expected unwanted effects of high doses, including hypertension and hypokalemia, must, however, be monitored. Oral doses up to 1500 mg of GR and 240 mg for intravenous injection have been employed [12,13].

The metabolism of GR from licorice extract has been investigated. Its processing starts in the oral cavity where it is partly broken down by glucuronidase, an enzyme in saliva, into glucuronic acid plus GA. In the intestine, GR is processed by bacteria-mediated hydrolysis and is mainly absorbed as GA and taken up into the liver where it is metabolized into glucuronide and sulfate conjugates. These conjugates are transported into the bile and released into the duodenum where they are again hydrolyzed to GA and subsequently reabsorbed, causing a pronounced delay in terminal plasma clearance. The intestinal metabolism means that the plasma concentration after oral GR is under the detection limit in rats and humans [14,15]. The pharmacokinetic profile of GR after intravenous injection shows a rapid distribution phase in different animal species [13]. In humans, the volume of distribution is 60 to 80 mL/kg [12,15], and the maximal plasma concentration after 200 mg GR is 80 µg/mL, corresponding to 100 µM, with a terminal half-life range of 3.5 to 9 h [12,15].

After an oral dose of 500 to 1500 mg GA in humans, Cmax in plasma was reached after about 4 to 6 h, ranging from 3.5 to 7 µg/mL (4 to 9 µM) [13]. Elimination was biphasic at doses above 500 mg [13] with a dose-dependent second elimination phase, respectively, 11.5 and 38.7 h after 1000 and 1500 mg [13]. The slow elimination of GA in humans likely reflects tissue binding. Individual plasma profiles in humans were compatible with enterohepatic cycling [13]. GA is eliminated as glucuronide or sulfate by the bile while urinary elimination is negligible, and the latter has an anti-inflammatory action [13,16].

## 3. Antiviral Effects 

The antiviral effects of a component of *Glycyrrhyza glabra* were first described more than 40 years ago by Pompei and collaborators, who observed that GR inhibited the growth and cytopathology of several unrelated DNA and RNA viruses [17]. Since then, numerous studies have reported that GR and GA can act against viruses belonging to different families [18].

In a range of concentrations from 0.025 mg/mL up to 6.7 mg/mL, GR and GA were reported active in vitro against different Herpesviridae, a large family of enveloped, double-stranded DNA viruses infecting animals and humans [17,19,20,21,22,23,24]. GR or aqueous licorice extracts inhibited the growth and replication of Herpes simplex virus type 1 (HSV-1), Epstein-Barr, Pseudorabies, and Varicella-zoster viruses (Table 2) [17,19,24]. Similar protective effects were observed in cells infected with the Paramixoviriade Newcastle or human respiratory syncytial viruses [17,25], or with the Rhabdoviridae vesicular stomatitis virus [17]. In these negative-strand RNA viruses, GR and aqueous licorice extract from 0.001 up to 6.7 mg/mL inhibited viral growth and increased IFN production. The growth and replication of enveloped, positive-strand RNA Flaviviridae in Japanese encephalitis, dengue, West Nile, and Yellow fever, were also reported to be inhibited by 0.1 to 2 mg/mL GR or 0.01 up to 6.7 mg/mL GA (Table 2) [26,27].

**Table 2:** Summary of the main studies on licorice extracts, GR and GA, against several unrelated DNA and RNA viruses.

Virus Family	Virus	Study		Compound Tested	Effective Concentration	Effects	Ref
*Hepadnaviridae*	Hepatitis A	In vitro PLC/PRF/5 cells		GR	0.25–2 mg/mL	Reduction of antigen expression and virus infectivity	[27,28,29]
		Humans Patients with acute autoimmune hepatitis		SNMC	200 mg/day i.v. x 4 weeks	Improvement of transaminases, prevention of disease progression	[30,31,32]
	Hepatitis B	In vitro Rat hepatocytes		GR	0.08 mg/mL	Suppression of transaminases	[33]
		PLC/PRF/5 cells		GR	Not known	Suppression of surface antigen	[33]
		PLC/PRF/5 cells		GR	1–2.5 mg/mL	Suppression of surface antigen	[32]
				GA	0.5 mg/mL		
		In vivo Guinea pigs		GR	3 or 67 mg/kg b.w. i.v	Suppression of surface antigen, reduction of transaminases	[34]
		Humans Patients with chronic hepatitis and liver cirrhosis		SNMC	120–160 mg/day i.v. 3 times/week for 4–36 months	Normalization of transaminases Reduction of viral load	[35,36]
		Patients with chronic hepatitis and acute exacer-bation		SNMC	200 mg/day i.v. × 5 days	Improvement of transaminases	[37]
	Hepatitis C	Humans Patients with chronic hepatitis		SNMC	80 mg/day i.v. × 4 weeks	Improvement of transaminases	[38]
		Patients with chronic hepatitis		SNMC	15 mg/day p.o. × 90 days	Improvement of liver function	[33]
		Patients with chronic hepatitis		SNMC	200 mg/day i.v. × 8 weeks	Improvement of transaminases and liver pathological features	[39]
		Patients with chronic hepatitis and liver cirrhosis		SNMC	120–160 mg/day i.v. 3 times/week for 4−36 months	Normalization of transaminases Reduced viral load	[40]
		Retrospective study on patients with chronic hepatitis		SNMC	200 mg/day i.v. × 8 weeks then 2–7 times a week × 2–16 years	Normalization of transaminases, reduced risk of hepatocellular carcinoma	[41]
		Patients with chronic hepatitis		SNMC	80 mg/day i.v. × 4 weeksor 200 mg/day i.v. × 8 weeks	Normalization of transaminases, reduced cirrhosis, reduced risk of hepatocellular carcinoma	[42]
		Phase I/II study in patients with chronic hepatitis		SNMC	200 mg i.v. 6 times a week × 4 weeks or 200 mg i.v. 6 times a week × 26 weeks	Improvement of biochemical and histological parameters, improvement quality	[43]
		Retrospective study on patients with chronic hepatitis		SNMC	200 mg/day i.v. 3 times a week × 4.3 years	Normalization of transaminases, reduced risk of hepatocellular carcinoma	[43]
*Herpesviridae*	Herpes simplex (HSV-1)	In vitro HEp2 cells		GR	0.8–6.7 mg/mL	Inhibition of virus growth	[17]
		Vero cells		GR	0.75 mg/mL	Inhibition of virus replication	[20]
		Vero cells		Aqueous G. glabra extract	2 mg/mL	Inhibition of virus entry	[21,22]
				Aqueous G. glabra extract Alkaline extract	0.3–3 mg/mL	Inhibition of virus growth	[22]
	Epstein-Barr	Raji cells		GR GA	0.7–3 mg/mL	Inhibition of virus growth	[23]
	Pseudorabies	Vero cells		GR	>0.3 mg/mL	Inhibition of virus growth	[19]
				GA	>3 mg/mL		
	Varicella-zoster	Vero cells		Diammonium glycyrrhizinate	0.025 mg/mL	Inhibition of virus replication	[24]
					0.005 mg/mL		
				GR in licorice powder extract	0.01–1.25 mg/mL	Inhibition of virus growth and infectivity	[19]
					0.125 mg/mL	Inhibition of virus growth	[24]
*Paramixoviridae*	Newcastle disease	In vitro HEp2 cells		GR	0.8–6.7 mg/mL	Inhibition of virus growth	[17]
					0.01–0.3 mg/mL	Inhibition of virus growth, increase in IFN production	
	Human respiratory syncytial	HEp-2 cells A549 cells		Aqueous G. uralensis extract GA	0.001–0.01 mg/mL	Inhibition of virus growth	[25]
*Rhabdoviridae*	Vesicular stomatitis	In vitro HEp2 cells		GA	0.8–6.7 mg/mL	Inhibition of virus growth	[17]
*Flaviviridae*	Japanese encephalitis	In vitro PS cells		GA	1–2 mg/mL	Inhibition of virus growth and replication	[26]
		Vero cells		GR	0.38 mg/mL	Inhibition of virus replication	
	Dengue	In vitro Vero cells		GA	0.01–0.1 mg/mL	Inhibition of virus replication	[44]
		Vero cells		GA	0.1–0.6 mg/mL	Reduction of infection	
	West Nile	In vitro Vero cells		GR	0.2 mg/mL	Inhibition of virus replication	[44]
	Yellow fever	In vitro Vero cells		GR	0.45 mg/mL	Inhibition of virus replication	[44]
*Orthomyxoviridae*	Influenza A	In vitro Hep2 cells		GR	0.8–6.7 mg/mL	Inhibition of virus growth	[17,45,46]
		MDCK cells		GR	0.25–1 mg/mL	Reduction of infectivity	[47]
		A549 cells MDCK cells HFL-1 cells		GR	0.4–0.8 mg/mL	Inhibition of virus replication	[48]
		MDCK cells		GR conjugated with aromatic amino acids methyl ester	0.2 mg/mL	Reduction of infectivity	[47]
				GR conjugated with S-benzyl-cysteine	0.2 mg/mL		
		In vivo Mice: C3H, ddY, CDF-1, C57BL, BALB/c, and athymic nude mice		GR	20 mg/kg b.w i.v	Reduction of infectivity	[48]
					50 mg/kg b.w. i.p.	Increased IFN production	
	Influenza and upper respiratory tract infection	Human hospitalized patients		GR	80 mg i.v infusion	Reduced hospitalization, body temperature lower 24 to 48 h after admission	[49,50]
*Coronaviridae*	SARS		In vitro Vero cells	GR	0.3–4 mg/mL	Inhibition of virus replication, adsorption, and penetration	[6,7]
			Vero cells fRhK-4 cells	GR	0.1 mg/mL>0.4 mg/mL	Inhibition of viral growth	
	Avian infectious bronchitis		In vitro Vero cells	GR diammonium	0.08–0.6 mg/mL	Inhibition viral growth	[7,51]
*Retroviridae*	HIV	In vitro MT-2, MT-4 cells		GR	0.1–1 mg/mL	Reduction of cellular and viral membrane fluidity; reduction of infectivity; inhibition of cell-to-cell	[45]
				GR	0.01–1 mg/mL	Inhibition of virus growth	
				GA	0.01 mg/mL		
		MT-4 cells		Aqueous *G. glabra* extract	0.3–0.5 mg/mL	Inhibition of virus growth	[52]
				Alkaline extract	0.2–0.05 mg/mL	Inhibition of virus growth	
		MT-4 cells		GR	0.4 mg/mL	Inhibition of virus growth	[22]
				GA	>0.5 mg/mL	Inhibition of virus growth	
*Reoviridae*	Porcino rotavirus	In vivo colostrum-deprived piglets, 3 days of age		Methanol *G. uralensis* extract	400 mg/mL p.o. 4 times/day	Cure of diarrhea, improvement of small intestinal lesions and fecal virus shedding, reduction of intestinalinflammation	[53]
*Arteriviridaein*	Porcine reproductive and respiratory syndrome	In vitro MARC-145 cells		GR	0.5–0.7 mg/mL	Reduction of virus penetration and proliferation	[54]
*Birnaviridae*	Infectious bursal disease	In vivo chicken: 24-days old brown chicks		Glycyrrhizinate dipotassium	20–80 mg/kg b.w. p.o. × 5 days	Increased immunological responses, increased IFN levels, inhibition of infection	[55]

SNMC = Stronger Neo-Minophagen C; HIV = human immunodeficiency virus; SARS = severe acute respiratory syndrome; IFN = interferon; *G. glabra* = *glycyrrhiza glabra*; *G. uralensis* = *glycyrrhiza uralensis*.

Studies have also investigated the effects of GR on Orthomyxoviridae, negative-sense RNA viruses causing influenza in animals and humans. In cells infected with influenza virus A, a pathogen causing influenza in humans, GR from 0.2 to 0.8 mg/mL reduced infectivity and inhibited viral replication [45,47]. In infected mice, intravenous GR, 20 mg/kg b.w., or intraperitoneal injection of 50 mg/kg b.w., lengthened survival and increased IFN-γ production [48]. In hospitalized patients with influenza and upper respiratory tract infection, the intravenous infusion of 80 mg GR reduced both the hospitalization stay and body temperature 24 h and 48 h after admission [49,50].

GR was reported to be effective in vitro from 0.08 to 4 mg/mL in inhibiting the replication, adsorption, and penetration of two SARS-coronaviruses (human SARS and the avian infectious bronchitis virus) [7,51]. In cells infected with human immunodeficiency virus type 1 (HIV-1) GR and aqueous licorice extracts were reported to inhibit viral growth [22,45,52].

GR’s action was partially ascribed to its ability to act as a saponin, reducing cellular and viral membrane fluidity. GR has a molecular structure similar to that of cholesterol, so it may easily diffuse across the lipid bilayer, disorganizing cholesterol-containing lipid rafts, which are important in the surface attachment of the virus to the cellular plasma membrane, thus suppressing cell-to-cell fusion induced by HIV [45].

Oral administration of a methanol-licorice extract to colostrum-deprived piglets infected with rotavirus cured diarrhea, reduced intestinal inflammation, and improved intestinal lesions [53]. GR from 0.5 to 0.7 mg/mL reduced virus penetration and proliferation in cells infected with porcine reproductive and respiratory syndrome virus, a positive-strand RNA Arteriviridae [54]. Oral treatment with 20 to 80 mg/kg b.w. glycyrrhizinate dipotassium to chicken with the viral infectious bursal disease was recently reported to stimulate immunological responses, raise IFN levels, and reduce viral load [55].

The main antiviral activity of GR investigated and described is against hepatitis viruses A, B, and C [27,28,29,30,31,32,33]. This stems from the observation in Japan, where licorice-derived compounds were traditionally used as antiallergic agents, that GR infused into patients suffering from allergic hepatitis had positive effects on transaminase levels [56]. GR and GA were then employed in clinical practice to treat liver diseases, notably chronic viral hepatitis. A pharmacological formulation called Stronger Neo-Minophagen C (SNMC), containing 0.2% (4 mg) GA as the main active constituent, 2% (40 mg) glycine, and 0.1% (2 mg) cysteine in 20 mL ampules, was developed and has been widely used in Japan and throughout East Asia over the past 30 years to treat chronic hepatitis B or C and cirrhosis (Table 2) [33,34,35,42]. Its use in the rest of the world is more restricted [33]. Clinical studies in patients with chronic hepatitis indicated that short- or long-term usage of SNMC is effective in reducing viral load, improving the liver histopathological features, and reducing transaminases, with no noteworthy side effects (Table 2) [28,33,34,35,36,37,38,39,40,41,42,43]. These properties result in a reduced risk of cirrhosis and hepatocellular carcinoma [43].

**Figure 2:**
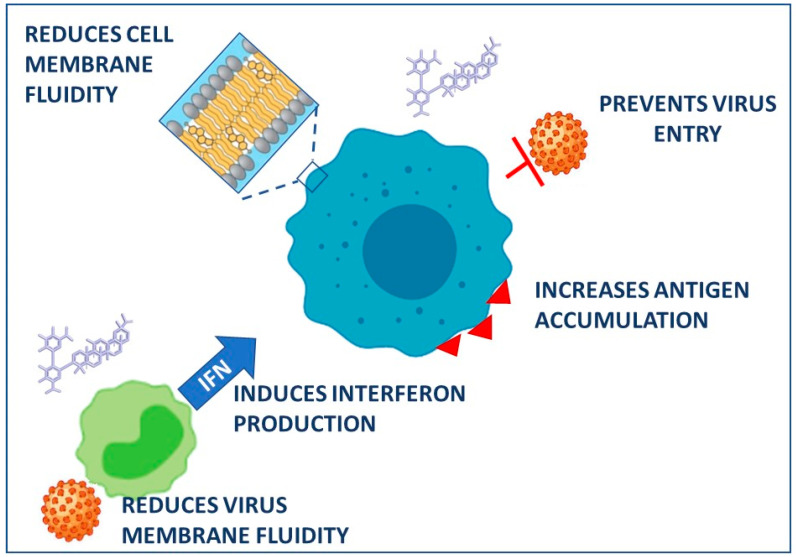
Proposed mechanisms in the antiviral effects of glycyrrhizin. Created with BioRender.com, accessed on 18 March 2021.

The mechanisms involved in the ability of GR to improve liver biochemistry and histology are not fully understood. Pharmacokinetic investigations indicate that the bioavailability of intravenous GR correlates with the functional capacity of the liver. In fact, the half-life and the total body clearance of GR were greater in patients with hepatitis than in healthy subjects [12]. GR dose-dependently inhibited the expression of hepatitis A virus antigen and reduced infectivity in the human hepatoma cell line PLC/PRF/5 without causing cytotoxicity [27] (Figure 2). GR also inhibited the entry of the virus into the cells, which occurs through receptor-mediated endocytosis [27]. This effect was ascribed to GR’s ability to interact with cell membranes, reducing both the fluidity and the negative surface charge (Figure 2). Experiments on isolated rat hepatocytes indicated that GR lowers the release of the enzyme aspartate aminotransferase and inhibits the activation of phospholipase A2, both involved in the cell membrane lysis [28].

In addition to the anti-viral and cytoprotective effects, GR and GA were found to display marked anti-inflammatory and immunomodulatory activities mediated by multiple mechanisms. Bacterial and viral infections activate the toll-like receptor 4 (TLR4) signaling pathway, leading to an increased production of pro-inflammatory cytokines, such as nuclear factor-κB, tumor necrosis factor, interleukin (IL)-1, and IL-6 [57]. GR and GA can interfere with the TLR4 activation and inhibit inflammation by different mechanisms. They may directly reduce the expression/activation of the TLR4 receptor thus suppressing cytokine production and inflammatory mediators. In addition, GR and GA may bind to proteins named high-mobility group box 1, directly activated by the TLR4 pathway to mediate the production and release of cytokines and chemokines [57]. Finally, GR and GA are also reported to increase the secretion of IFN, helping the prevention of viral attachment [58]. All these activities resulted in the improvement of the immunological status of patients with hepatitis B and the increase in the antigen’s immunogenicity [31] (Figure 2). Based on the findings in mice infected with influenza A virus [48], it was hypothesized that GR may protect against the damage caused by hepatitis viruses by inducing the production of IFN, causing cytokines secreted by infected cells of the immune system to respond to infectious agents. All these findings supported the potential use of GR in patients with hepatitis who do not respond to IFN therapy.

## 4. Effects on COVID-19 Infection

The antiviral activities of GR in general and specifically for SARS-CoV, suggest its potential use in treating COVID-19. In 2003, Cinatl et al. showed that GR hinders the internalization of the SARS-CoV-1 virus in a concentration-dependent manner, with an IC_50_ of 300 µM [6]. Though the mechanism of action is not clear, it seems that GR is a multi-targeting compound, interacting with various viral and cellular processes important for viral internalization and replication [57,59].

**Figure 3:**
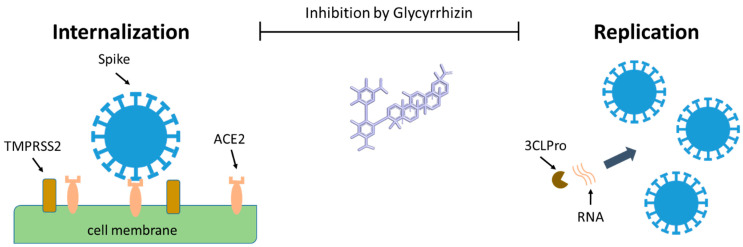
The possible effects of glycyrrhizin on the internalization of the SARS-CoV-2 virus in the host cell, and its replication. Created with BioRender.com, accessed on 18 March 2021.

The internalization of the SARS-CoV-2 virus to the host cell starts with the viral spike protein binding to the cellular receptor angiotensin-converting enzyme 2 (ACE2) (Figure 3) [60]. Once bound to the cell surface of the host, via ACE2, the spike protein is then cleaved by the host transmembrane serine protease 2 (TMPRSS2) into the S1/S2 domain. The S1 domain contains the receptor-binding domain (RBD) which binds directly to ACE2. After cleavage of the spike protein, the virus is internalized by the cell by viral and cellular membrane fusion.

This step is followed by the expression and replication of the viral genomic RNA which are incorporated into newly produced viral particles. Replication is mediated by several proteases, and 3-chymotrypsin-like cysteine protease (3CLpro or MPro) enzyme is one of the key players sustaining the viral life cycle [60]. The newly formed virions are then transported to the cell surface and released by exocytosis into the extracellular space (Figure 3).

Several in silico docking studies suggested a direct interaction of GR and GA to these key players in virus internalization and replication: ACE2 [61,62,63], spike protein and its RBD [63,64,65,66], and 3CLPro [63,65,67,68,69,70,71,72,73,74]. Only a few in silico analyses were negative (3CLpro [68,75], Spike [76]).

The main protease 3CLpro contains several canonical binding pockets, denoted P1-P4, in its active site [77]. In silico studies suggest the binding of GR to these pockets [63,67,68,69,70,71,72,73,74]. However, when tested on enzyme activity, the results were contradictory. GR was not present among the active compounds in a large-scale high-throughput in vitro study (i.e., it did not inhibit the enzyme activity by 60% at a concentration of 50 µM) [77], though a recent study showed a 70% inhibition of the enzyme activity with 30 µM GR [78]. The same authors showed that the IC_50_ of GR in a Vero E6 cell infection assay was around 500 µM, possibly suggesting limited internalization of GR.

Mahdian et al. suggested different potential binding pockets for GR, both on the spike protein (on RBD and in another domain close to the S1/S2 cleavage site), and on ACE2 (in a domain close to the interface where RBD binds) [63]. GR binding to RBD and to the S1 spike subdomain was also suggested by Yu et al. based on in silico data [64]. These authors, however, provided experimental support too, although not so definite. They employed surface plasmon resonance (SPR), a technique widely used to study interactions between unlabeled molecules and determine their binding constants [79]. SPR indicated relatively high-affinity binding of GR to the S1 domain (Kd 0.87 μM) [64], while a binding inhibition assay based on nanobeads showed more than 20 times lower affinity (IC_50_ 22 μM). Another study investigated the GR binding to the S2 domain and ACE2, in silico with positive docking results, and experimentally by SPR [80]. In this case, however, the affinity estimated by SPR was very low (Kd more than 2 mM) suggesting a nonspecific interaction. 

The interaction of GR with ACE2 was also investigated [62]. SPR studies indicated a Kd of 4.4 µM, but the enzymatic activity of ACE2 was not affected, suggesting that it does not bind to the catalytic domain [62]. The docking results did indicate that GR binds to a domain of ACE2 close to the binding site responsible for the interaction with RBD, thus suggesting that it might interfere with the spike protein-mediated interaction of the virus with the host cells. However, this has not been demonstrated experimentally: Xu et al. did not observe any effect of GR, up to 100 µM, on viral internalization in HEK293T cells [81], in line with the results shown by Sand et al. in Vero E6 cells (IC_50_ 500 µM) [78].

On the basis of the findings by these different authors, indicating that GR activity varied by some orders of magnitude, one can hypothesize that the molecule has to penetrate the cell to reach a reasonably high concentration to be effective.

Another mechanism by which a compound might hinder viral internalization is the reduction of ACE2 on the cell surface. GR inhibits the expression of ACE2 due to a reduction in the signaling activity of the high mobility group box 1 complex in a dose- and time-dependent manner [62]. GR may also reduce SARS-CoV-2 infectivity by inhibiting the lipid-dependent attachment of the virus to host cells, as for other viruses [57]. However, no experimental data are available to support this hypothesis.

Recent evidence suggests that TLR4 can strongly interact with the spike protein of SARS-CoV-2 and induce an inflammatory signaling pathway which results in an increased expression of ACE2 [82]. It cannot be excluded that GR and GA may prevent viral infection by blocking TLR4 and thus ACE2 expression [82].

Even though many in silico studies suggest that GR interacts with proteins involved in SARS-CoV-2 virus infection and reproduction, supporting in vivo and in vitro data are scarce, even more so for GA, the main GR metabolite. There is only one in silico study suggesting GA binding to the protease 3CLPro [69].

GR and GA are also included in the ReFRAME library [83], a library of 12,000 compounds used for drug repositioning screening. When these compounds were tested at a concentration of 5 µM for inhibition of viral infection in Vero E6 cells, GR and GA were not among those active [84,85].

The interest in licorice extract to prevent or treat COVID-19 is also justified by its anti-inflammatory and antiallergic actions [57,82,86], which have been attributed to the corticosteroid-like activity of GR and GA [11]. Licorice has been employed to treat dry cough and chronic obstructive lung diseases due to GR’s ability to reduce tracheal spasm induced by histamine, and to its antitussive and expectorant properties [11].

Based on these findings and the known ability of GR and GA to boost IFN secretion, with anti-inflammatory effects, two clinical trials have been launched on the effects of licorice extracts on clinical symptoms in COVID-19 patients. One is a pilot, single-center, non-randomized trial, registered on ClinicalTrial.gov (NCT04487964), testing the effects of licorice extracts administered together with Boswellia serrata gum as complementary medicine in Egyptian patients with COVID-19. In addition to conventional therapy, patients receive one capsule twice daily containing 250 mg of standardized licorice extract, corresponding to 62.5 mg GR, for 10 days, and 2 g four times daily for 15 days of Boswellia gum resin. The other is a single-center, open-label, randomized clinical trial with parallel-group design, registered in the Iranian Registry of Clinical trials (IRCT20200506047323N2) which will look at the effects of licorice root extracts in moderately ill patients with pneumonia from COVID-19 [87]. In addition to the standard treatment, D-REGLIS tablets containing 380 mg of standardized dry extract of licorice with less than 3% GR at a dose of 760 mg were given three times a day for 14 days. No results have been published yet.

## Figures and Tables

**Table 1:** Chemical structures of compounds in licorice root extracts with their most widely used names and CAS registry number. Data retrieved from PubChem.

Glycyrrhizin (Glycyrrhizic acid) CAS: 1405-86-3	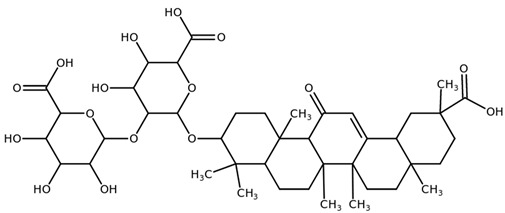
Glycyrrhetinic acid (Enoxolone) CAS: 471-53-4	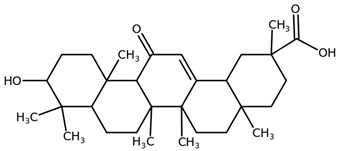
Isoliquiritigenin CAS: 961-29-5	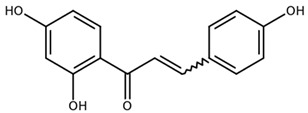
Licochalcone A CAS: 58749-22-7	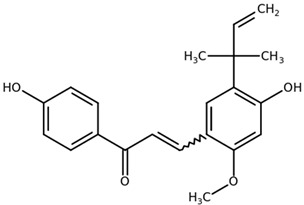
Liquiritigenin CAS: 578-86-9	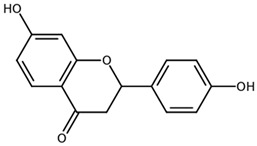
Liquiritin CAS: 551-15-5	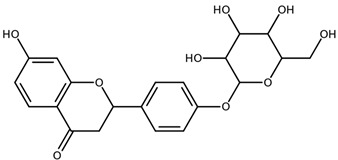
Licoflavone B CAS: 91433-17-9	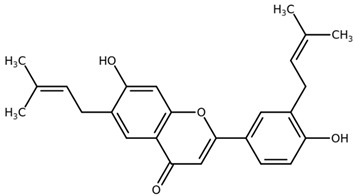
Glisoflavone CAS: 125709-32-2	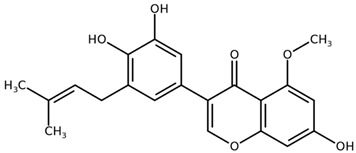
Isoangustone A CAS: 129280-34-8	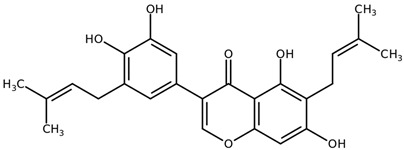
Semilicoisoflavone B CAS: 129280-33-7	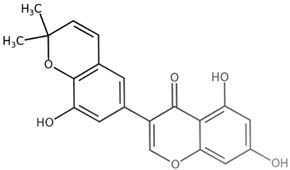
Shinpterocarpin CAS: 157414-04-5	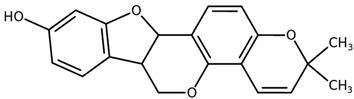
1-Methoxyficifolinol CHEBI: 69096	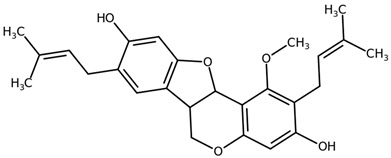
Licoriphenone CAS: 129280-36-0	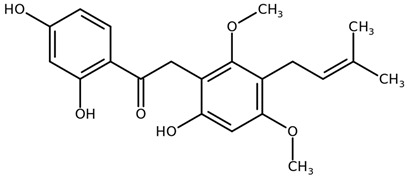
Glabridin CAS: 59870-68-7	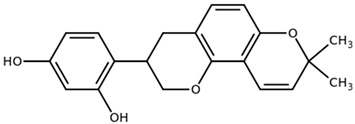
Glabrene CAS: 60008-03-9	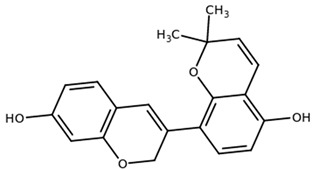
Kanzonol R CAS: 156250-73-6	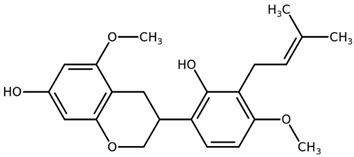
Licopyranocoumarin CAS: 117038-80-9	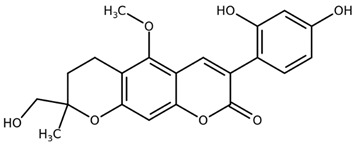
Licoarylcoumarin CAS: 125709-31-1	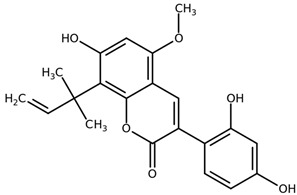
Licocoumarin A	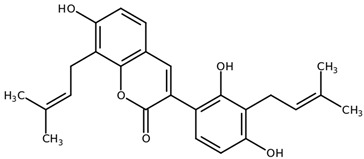
Tetramethyl pyrazine-2,3,5,6-tetracarboxylate CAS: 35042-21-8	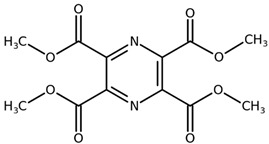
Alpha-terpineol CAS: 98-55-5	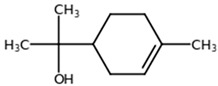
Furfural CAS: 98-01-1	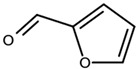
2,3-Butanediol CAS: 513-85-9	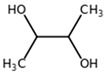

## Data Availability

This is a Review of the data available in literature. No data were analysed or generated during the study.

## Abbreviations

GR	Glycyrrhizin
GA	Glycyrrhetinic acid
*G. glabra*	*Glycyrrhiza glabra*
*G. uralensis*	*Glycyrrhiza uralensis*
HIV	Human immunodeficiency virus
HIV-1	Human immunodeficiency type-1 virus
SARS	Severe acute respiratory syndrome
SNMC	Stronger Neo-Minophagen C
HSV-1	Herpes simplex virus type 1
IFN	Interferon
ACE2	Angiotensin-converting enzyme 2
TLR4	Toll-like receptor 4
IL	Interleukin
TMPRSS2	Transmembrane serine protease 2
RBD	Receptor binding domain
3CLpro	3-chymotrypsin-like cysteine protease
SPR	Surface Plasmon Resonance
GRAS	Generally Recognized as Safe
FDA U.S.	Food and Drug Administration
